# Knowledge, Attitudes and Intention to Donate Organs among the Public, Health Sciences Students and Health Personnel: A Scoping Review with a Systematic Review of Malaysian Studies

**DOI:** 10.21315/mjms2024.31.1.16

**Published:** 2024-02-28

**Authors:** Emad Eldin Naguib Saleh, Jin Wei Tan, Hui Bing Lim, Oppilal Annamalai, Ming Sim Chii, Sherreen Elhariri

**Affiliations:** 1 Department of Obstetrics and Gynaecology, International Medical University, Clinical Campus, Johor, Malaysia; 2 International Medical University, Negeri Sembilan, Malaysia; 3 Department of Surgery, International Medical University, Negeri Sembilan, Malaysia

**Keywords:** organ donation, knowledge, attitude, intention

## Abstract

**Background:**

Various barriers lead to a shortage of organs for transplantation in Malaysia. One drive to improve the organ donation rate operates through future healthcare practitioners and practitioner advocacy. This scoping review was carried out to establish and summarise findings about organ donation-related articles among the public, health sciences students and health personnel. A further aim was to synthesise the latest data on knowledge and attitudes towards organ donation in the Malaysian population.

**Methods:**

PubMed, Scopus, Google Scholar and the Malaysian Medical Repository (MyMedR) were used for a search conducted up to May 2022. Relevant search terms included ‘Organ donation’ and ‘Malaysia’. Journal articles related to knowledge, attitudes and intention were grouped under the general public and health science. Students and health personnel were included. Eligible studies were reviewed by two independent reviewers. Any disagreements were resolved by consensus with a third reviewer.

**Results:**

The 31 included articles revealed an increased level of awareness among the public regarding organ donation. The analysis identified that nonrecognition of brainstem death (38.5%), no knowledge of how to contact the Organ Transplant Coordinator (82.3%) and never approaching the families of a potential donor (63.9%) led to a lack of confidence among healthcare practitioners to promote organ donation.

**Conclusion:**

The shortage of organ donors is the result of the failure to identify the expected donor, obtain consent and procure the organs due to the passivity of Malaysian health professionals in promoting the organ donation process.

## Introduction

Most countries in the world face a shortage of organs required for transplantation and Malaysia is no exception. With only 0.48 donations per million people in 2010, Malaysia has one of the lowest deceased organ donation rates, along with countries like Myanmar (0.02) and Guatemala (0.52), compared to countries such as Spain (34.13) and Belgium (25.61) (1). From 1997 until 2006, only 162 procurement procedures were carried out in Malaysia, resulting in a need for organ transplants that greatly exceeds the supply that is donated (2). Certain communities are unwilling to consent to organ donation for multiple reasons. In general, Asians are more reluctant than Caucasians to donate their organs, and within Asian communities, several cultural and religious factors contribute to disparities in organ donation (3). This naturally applies to Malaysia, a multi-ethnic, multicultural and multireligious Asian country.

Ethnicity, culture and religion are recognised as some of the greatest obstacles to organ donation in Malaysia, despite organ donation advocations from religious leaders and popular figures (3). However, a recent study showed that the shortage of organ donors is the result of the failure to identify them, obtain consent and procure the organs due to passivity among health professionals in promoting the organ donation process (4). The study identified that nonrecognition of brainstem death (38.5%), no knowledge of how to contact the Organ Transplant Coordinator (82.3%) and never approaching the families of a potential donor (63.9%) had led to a lack of confidence in healthcare practitioners to promote organ donation. The authors thus concluded that continuing medical education and increasing the knowledge of medical professionals regarding organ donation was key to improving the rate of organ donations from deceased donors in Malaysia.

While multiple studies on the knowledge and attitudes of healthcare workers towards organ donation in Malaysia exist, they have yet to be reviewed. The aim of this systematic review was to synthesise the latest data on knowledge and attitudes towards organ donation among the public, health sciences students and health personnel, in hopes that it can serve as a baseline for future intervention-related research.

## Methods

Scoping reviews are conducted for the purpose of identifying knowledge gaps, scoping a body of literature, clarifying concepts or investigating research conduct. Scoping reviews may also be helpful precursors to systematic reviews and can be used to confirm the relevance of inclusion criteria and potential questions (5).

A comprehensive search was conducted according to Preferred Reporting Items for Systematic Reviews (PRISMA) guidelines, using the scoping review methods proposed by Arksey and O’Malley (6) to map the findings regarding organ donation-related articles among the public, health sciences students and health personnel. A further aim of this systematic review was to synthesise the latest data on knowledge and attitudes towards organ donation in the Malaysian population using the PubMed, Scopus, Google Scholar and the Malaysian Medical Repository (MyMedR) databases. MyMedR was developed as an open-access database focused on health- and biomedical-related research conducted in Malaysia and incorporates data from PubMed and MyJurnal (https://myjurnal.mohe.gov.my/public/browse.php). Only articles written in the English language were used. The terms used for searching to identify articles about the research questions were as follows: (‘organ donation’ OR ‘organ transplantation’) OR ‘knowledge’ OR ‘attitude’, OR (‘intention’, ‘practice’, ‘willingness’) and Malaysia. Only journal articles were selected and the searches were confined to dates up to May 2022.

The screening was completed by two independent reviewers who reviewed articles that fulfilled the inclusion criteria. The inclusion criteria were original journal articles based on knowledge, attitude, perception (KAP) and intention regarding organ donation conducted in Malaysia. Relevant studies were input into Microsoft Excel for data management. The study titles and abstracts were screened manually by the same independent reviewers with the inclusion and exclusion criteria identified; any dispute was resolved by a third reviewer through mutual consensus. For the removal of duplicated articles, we used the EndNote20 reference management tool and then checked deduplication manually by comparing the digital object identifier (DOI) numbers and abstracts. Organ donation, as discussed within this review, included living donation, deceased or cadaveric donation and tissue donation. The process of the search, inclusion and exclusion was presented using a standardised PRISMA 2020 flow diagram. Quality assessment was not performed, as the purpose of this review was to identify the availability of studies related to knowledge, attitude and intention among identified population groups in Malaysia, rather than excluding studies based on quality assessment.

### Data Extraction

The full-text articles remaining after screening were reviewed manually, and the data extraction process was conducted by formulating extraction points on Microsoft forms by two independent reviewers performing data entry and compiling them into Microsoft Excel for review and data organisation. Any conflicts arising from data extraction were then reviewed by a third member to settle on a mutual consensus, as discussed with the two initial reviewers. The extracted data were then filtered, screened, summarised and presented in a tabulated form, as described below, and were divided into respective population groups in the following order: general public (Table 1), health science students (Table 2) and healthcare personnel (Table 3).

## Results

### Study Selection

**Figure 1:**
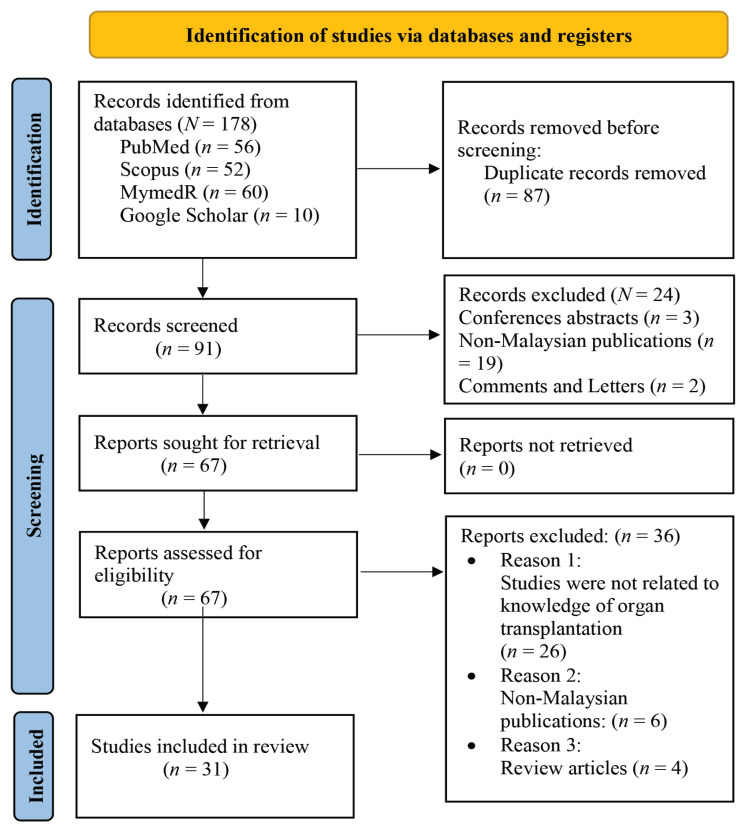
PRISMA 2020 flow diagram for new systematic reviews

The flowchart of the article selection process is documented and described in Figure 1. A total of 178 articles were identified from the search results. Of those, 87 articles were excluded as duplicates, 91 articles were screened and 24 studies were excluded, as the records were conference abstracts (*n* = 3), non-Malaysian publications (*n* = 19) or comments or letters (*n* = 2). For full-text assessment, 36 articles were excluded, as 26 of them were not related to organ donation KAP, 6 were non-Malaysians articles and 4 were review articles.

A total of 31 articles that met the inclusion criteria were ultimately included. The articles were categorised under the following population groups: the public, health personnel and health science students, and tabulated accordingly. The first article about the public was published in 1998 and the first related to health personnel was published in 2000. Most of the articles were published after 2011. The locations in which the research was conducted were identified largely as Kuala Lumpur and the Klang Valley (*n* = 20) and most studies had a cross-sectional study design (*n* = 30) or an overshadowing interventional study design (*n* = 1).

## Synthesis of Results

The articles searched were categorised based on the population of interest: the public (*n* = 18), health science students (*n* = 6) and healthcare personnel (*n* = 7). Under the general public category, all studies that assessed knowledge, attitude and intention to donate employed a cross-sectional study design and used self-reporting questionnaires. Most of those studies were conducted in Peninsular Malaysia, in Kuala Lumpur (*n* = 11), rather than in East Malaysia, in Sabah (*n* = 1) and Sarawak (*n* = 1). The study settings were largely within the community (*n* = 10), followed by tertiary healthcare facilities (*n* = 4), Muslim communities (*n* = 2), primary healthcare centres (*n* = 1) and schools (*n* = 1).

In the healthcare personnel category, we found six studies that assessed the knowledge, attitude and intention to donate. These included cross-sectional studies (*n* = 5) utilising self-reporting questionnaires and an interventional study (*n* = 1) that included educational material between a pre- and post-test. All studies (*n* = 6) were conducted in Kuala Lumpur, with the interventional study conducted in additional states, including Selangor, Pahang and Kelantan.

In the health science student category, the available studies (*n* = 7) were conducted in individual health science-related tertiary education centres.

## Discussion

### Knowledge, Attitude and Practice Regarding Organ Donation among the Public

More studies were focused on the KAP among the general public than among healthcare professionals or health sciences students. The reason for this could be a link to a targeted outcome, which is an increase in organ donation rates. We noted that most of the identified papers employed a cross-sectional study design. No interventional studies were conducted to improve the KAP of organ donation. While these cross-sectional studies conducted on the public population help in understanding the barriers to organ donation, an attempt to overcome or counter these barriers through an interventional study might be helpful. Interventional studies would also allow us to determine whether our chosen methods of intervention are beneficial in achieving our target outcome of increased organ donation rates.

We also noted that many of the studies focused on the population within Kuala Lumpur and Selangor. While this may reflect the large number of universities, healthcare facilities and researchers in these areas, we need to exercise caution as the cumulative data may not be an accurate representation of the whole country. We also noted differences in the questionnaires used in assessing KAP. Even though these questionnaires had been validated through pilot studies, we cannot conduct a fair comparison of data obtained at different time points or locations if they have been collected using different questionnaires. Stratification of data collection and analysis tools, including having a standardised questionnaire, is required for use in different KAP studies on organ donation at various locations and times of study.

In general, the majority of studies revealed that most of the population is aware of the existence of organ donation. However, the rate of organ donation has been relatively stagnant over the years, most likely due to a lack of knowledge and information, which were identified as the main barriers in most of the studies. Overall, brain death was the least known subject relating to organ donation among the general population (7, 8) and could be related to the rejection of organ donation by family members of brain-dead patients. Another significant barrier identified was that many persons who were willing to be organ donors did not know how to register as organ donors. The result is that many potential organ donors are not registered (2). We also need to identify the common false beliefs about organ donation in the community and provide education on these issues (9).

These evaluated studies revealed an increased level of awareness among the public regarding organ donation. However, efforts to educate the public on organ donation have been too superficial and inadequate to convince many to pledge as donors. Should we organise promotional campaigns for organ donation in the future, we could choose to focus on the concept of brain death as the main discussion topic. Most studies have shown that lower educational levels, Muslim religion and poor knowledge of organ donation were the main factors that reduced a person’s willingness to donate.

The major religion in Malaysia does not reject the idea of organ donation. However, studies have suggested that many people fear organ donation because they are unsure whether it is permissible in their religion. In 2014, a study by Tumin et al. (10) conducted on the Muslim population identified that almost half the respondents believed that organ donation was prohibited in Islam and may be contrary to Islamic principles. A way around this issue could be to involve religious leaders as educators on organ donation and to clarify the stance of their religion surrounding this topic.

One study also indicated that the provision of incentives, regardless of the significance of the amount, does not motivate the community to opt for living organ donation. The main barrier to living organ donation remained consistent; for example, in kidney donation from a living person, the main barrier was a lack of knowledge that donors could live with one kidney (11). Intervening by providing education on the effect of donating one kidney, the expected quality of life, life expectancy, and how to register as a living kidney donor might improve general public KAP regarding living organ donation. Hence, future studies could focus on interventional trials to promote knowledge and understanding of organ donation.

In 2015, a study by Tumin et al. (12) explored the attitudes of family members of dialysis patients towards deceased organ donation under the informed consent system and presumed consent system. This revealed that respondents were more willing to donate their organs under the informed consent system than under the presumed consent system. Hence, although the presumed consent system seems to have higher organ donation rates in countries that apply it, an important point to note is that the general public in our country does not demonstrate an attitude of readiness towards it (12).

### Knowledge, Attitude and Practice Regarding Organ Donation among Health Sciences Students

A general assumption is that greater knowledge regarding organ donation would lead to a better attitude among health science students. The aim of this scoping review was to identify the extent of KAP towards organ donations among health science students from Malaysia based on the available research articles. These included cross-sectional studies that were conducted in various medical and health science universities, including some of the renowned universities, such as University of Malaya, Management and Science University, Melaka Manipal Medical College Melaka and Universiti Kuala Lumpur-Royal College of Medicine Perak. The data were collected through self-administered questionnaires. Our expectation was that the population of health science students would have a better knowledge and attitude about organ donation compared to the general population, as they would be familiar with the concept of the shortage of donor organs in the country and would be more open-minded regarding organ donation as they belong to the modern generation. The KAP of general organ donation, including cadaveric, eye and kidney donations, was assessed among health science students.

Regarding knowledge, a study by Haque et al. (13) concluded that more than half of the students had good knowledge about organ donation, while the remainder had mostly average knowledge and a small portion had poor knowledge. In this population, medical students seemed to have better knowledge compared to radiology and pharmaceutical students. This might be due to exposure to the idea of liver and renal transplantation in cases of end-stage renal failure and liver failure among the senior medical students as a part of their clinical exposure, which the other health science students lack. However, a study by Bharti et al. (14) showed that most students seemed to have obtained transplant knowledge from the mass media or newspapers.

Similarly, according to a study by Tumin et al. (15) in 2016, medical students were more willing than nursing students to donate their organs after death. Overall, approximately half of the population of students appeared willing to donate their organs after death and a minority of them were already registered as organ donors. The most common reason given by those who refused to become organ donors, according to a study by Jun (16), was the lack of information available about organ donation, followed by not having decided about becoming a pledger. Muslim students were markedly less likely to agree to organ donation compared to students of other religions. Tumin et al. (17) noted that non-Muslims were 1.5 times more willing to donate their organs than Muslims. This might be due to the stigma against organ donation that existed within the Islamic community earlier, when there was a misconception that organ donation was against their religion. However, recent advances on this topic have clarified that organ donation is indeed not against the Islamic religion. This fact must be spread more widely among the Islamic communities so that false information does not mislead individuals who might otherwise be willing to donate their organs, given the fact that it could potentially be lifesaving. Jun (16) also showed that higher educational qualifications among the parents of the students led to better attitudes and willingness towards organ donation.

While most of the studies showed that increased knowledge contributed to increased willingness, surprisingly, one study by Keya et al. (18) showed no association between knowledge and attitude towards organ donation. This result indicates that greater knowledge alone does not assure more willingness to donate and that various other factors, such as cultural aspects, personal desire and economic status, contribute to the willingness to donate organs. Even though not much can be done in terms of the cultural aspects, personal desires and economic status of health sciences students, the promotion of the importance of organ donation and the increasing need for organ donation should be advocated by governmental and non-governmental organisations to improve the rate of organ donation. Interventional studies using promotional speeches and videos would be useful for identifying effective interventions. Implementing the identified interventions on the target population would then boost organ donation rates.

### Knowledge, Attitude and Practice Regarding Organ Donation among Healthcare Personnel

Our initial search indicated that the shortage of organ donors is a result of a lack of knowledge regarding organ donation, which has led to passivity among healthcare professionals in promoting the organ donation process (19). Our findings, after thorough research, support this hypothesis, with respondents admitting that organ donation rates are low because of a lack of counselling by healthcare personnel (20). The cross-sectional studies provided an accurate representation of the healthcare practitioners’ opinions before the study, while the interventional study demonstrated the relationship between knowledge and willingness to donate of the respondents before and during the study. There was no focus on any specific type of organ donation. The studies were mostly done in hospitals in Kuala Lumpur, the capital of Malaysia, where the population is more educated and open to the idea of organ donation. This may have led to a bias in the results; therefore, the findings may not be an accurate representation of all healthcare practitioners in Malaysia. Nevertheless, this does not dismiss the validity of these studies, as they represent a large percentage of Malaysian healthcare practitioners. Based on the reviewed studies, doctors are more knowledgeable and more easily convinced of brain death than nurses (21). The level of knowledge is an important factor, as it predicts the healthcare professional’s attitude toward organ donation. This notion is supported by the fact that doctors have a more positive attitude towards organ donation and are more willing to donate than nurses (20).

An interventional study by Shukeri et al. (22) identified improved attitudes of healthcare professionals towards organ donation after an educational website-based intervention, as they were more willing to donate their own as well as their relatives’ organs post-intervention. The study also proved that increased knowledge among healthcare professionals solved the problem of their passivity in approaching families of potential donors and informing the transplant teams. This was an issue identified in the other studies but was alleviated after the healthcare professionals received more information and became more comfortable with counselling patients and relatives regarding the subject of organ donation. It is refreshing to know that most doctors and nurses support organ donation and believe that it can save lives.

However, support for organ donation did not necessarily translate into willingness to donate, as one study found that only half of those supportive of organ donation were willing to donate their own organs (20). The reasons provided mainly consisted of religious beliefs or their own beliefs, despite the claims by most respondents that their religion does not forbid organ donation. Willingness to donate also varied from one study to another, ranging from 47.8% to 68.0%. One study also found that among those willing to donate their organs, only a third carried donor cards at the time of the study (20).

Many healthcare professionals are also against implementing a presumed consent system despite supporting organ donation. Tumin et al. (23) found that almost half (45.8%) of the surveyed healthcare professionals objected to donating their organs if a presumed consent system was in place, with most of them who were initially unwilling to donate were from the Malay ethnic group or had incomes of RM3,000 or lower. Malaysia is not ready to move towards a presumed consent system if healthcare professionals are not keen on the idea, as they are a major factor in organ donation advocacy. More studies should be done to understand this pattern to identify solutions and raise confidence in the organ donation system (23).

Many issues faced by Malaysian healthcare professionals have been identified in this review. Despite being knowledgeable about this subject, some healthcare professionals are still indecisive when it comes to donating their own organs (24). This may reflect contributions from other factors, such as religion and their own beliefs. Perhaps more factors can be identified with more studies on our healthcare professionals. Only with a deeper understanding of their concerns can we resolve each issue individually. For example, the interventional study by Shukeri et al. (22) provided us with proof that educating healthcare professionals is effective in increasing confidence in our organ donation system. Thus, more interventional studies should be encouraged and education on organ donation should be prioritised in medical and nursing schools to produce doctors and nurses who are well-informed, as our findings proved that more knowledgeable healthcare professionals are more likely to advocate organ donation to their patients and families. Although there is much room for improvement, it is encouraging to know that Malaysian healthcare professionals are progressing forward and accepting new information while correcting old ideologies. Advocation efforts and campaigns for organ donation have shown fruitful results and should be continued, as organ donation is a lifesaving opportunity.

## Conclusion

Our study revealed an increased level of awareness among the public regarding organ donation. However, several cultural and religious factors contribute to disparities in organ donation in Malaysia, and most studies have shown that lower educational levels, Muslim religion and poor knowledge of organ donation were the main factors that reduced willingness to donate. The shortage of organ donors is the result of the failure to identify the expected donor, obtain consent and procure the organs, largely due to passivity among healthcare professionals in promoting the organ donation process. Thus, continuing medical education and nourishing the knowledge of medical professionals on organ donation are the keys to improving the rate of organ donations from deceased donors in Malaysia.

## Figures and Tables

**Table 1:** Summary of included studies for knowledge, attitude and intention to donate amongst the public

Author(s); Article title; Year	State	Study aim	Study design	Study setting	Results
Tumin et al.; Strategies targeted at motivating unrelated living kidney donation; (2013) (9)	Kuala Lumpur	To assess the willingness of Malaysians with post-secondary education to be living kidney donors.	Cross-sectional	Community	The findings demonstrate that out of 688 respondents, only 198 (28.8%) were willing to become living organ donors. The main reasons were associated with a lack of knowledge of the medical issue. The three main reasons for those who were not willing to be donors were: i) 212 (37.4%) were not convinced that humans could live with one kidney, ii) 112 (19.8%) perceived that they were not medically fit, followed by iii) 85 (15%) that feared surgery.
Zumin et al.; Factors that hinder organ donation: religion-cultural or lack of information and trust; (2013) (3)	Kuala Lumpur	To assess factors that influence Malaysians with tertiary level of education on their willingness to donate organs.	Cross-sectional	Community	The findings demonstrate that out of 688 respondents, 351 (52.5%) were willing to be organ donors upon death. The main reasons for those who were not willing to be donors were associated with a lack of trust and information, 30.1% were not convinced that their body part would be used beneficially, and 29.2% felt that they did not have enough access to information.
Tumin et al.; Organ donation in Muslim countries: the case of Malaysia; (2013) (25)	Kuala Lumpur	To assess factors influencing Malaysian Muslims’ decision to become deceased organ donors.	Cross-sectional	Muslim community	This study shows that out of 779 respondents, 508 (65.2%) rejected to be deceased organ donors. The findings showed that lack of access to information (27.7%) and not being convinced that their body part would be used beneficially (27.5%) were the main reasons for respondents to reject being an organ donor. For those respondents that were willing to be organ donors but have not registered, 37.5% claimed they did not know the procedures for registration as an organ donor and 24.1% have no motivation to register as a donor.
Tumin et al.; Living kidney donation: the importance of public education; (2014) (11)	Kuala Lumpur	To assess the factors that influence Malaysian’s willingness to become living kidney donors.	Cross-sectional	Community	Out of 1,310 respondents, 945 (72.1%) rejected to be living kidney donors even if the government comes up with a policy to reward them with a huge amount of incentive. The three main reasons for not being willing to be a donor were: i) they were not convinced that humans could live with one kidney (35.6%), ii) they were not medically fit (23.3%), iii) followed by fear of surgery or operations (14.3%). The main reasons for those willing to become a living kidney donor included, doing something noble in life (50%) and the personal urge to help kidney patients (24.9%).
Tumin et al.; Do family members of dialysis patients have a positive attitude towards organ donation?; (2015) (12)	Kuala Lumpur	To explore the attitude of family members of dialysis patients towards deceased organ donation under the informed consent system and presumed consent system.	Cross-sectional	Community	Under the informed consent system, 181(51.8%) out of 350 respondents were willing to donate organs upon death. Out of the 181 respondents, 23 were registered donors, 23 of them were willing to register during the survey, while the others were not ready to register during the time of the survey. Under the presumed consent system, 202 (57.7%) out of 350 respondents would sign an objection. This included 150 (88.8%) out of the 169 respondents who were unwilling to donate their organs under the informed consent system and 52 (28.7%) out of 181 respondents who were originally willing to donate their organs under the informed consent system.
Lim et al.; Factors influencing attitude toward organ and tissue donation among patients in primary clinic, Sabah, Malaysia; (2020) (7)	Sabah	The study assesses the knowledge of and factors influencing attitudes toward organ and tissue donation among patients in a primary clinic.	Cross-sectional	Primary health care clinic in Kota Kinabalu, Sabah	The study showed that about 55.6% of the participants had good knowledge about organ donation based on questions such as definition, the age limit for organ donation, causes of organ transplantation, and organs and tissues which can be donated in Malaysia. Based on knowledge of brain death 32.6% of them have good knowledge and 67.4% had poor knowledge, 50.9% of the participants had a positive attitude toward organ donation and the remaining had a poor attitude toward organ donation. It was revealed that 48% of Bumiputra respondents had a positive impression of organ donation. Participants with lower educational levels and monthly income of < RM2,000 had poorer attitudes. It was also seen that 69% of respondents with good knowledge had a positive attitude toward organ donation, whereas 72% of respondents with poor knowledge had a negative attitude.
Lim et al.; Attitudes of parents of school going children in Federal Territory of Kuala Lumpur to kidney donation; (1998) (26)	Kuala Lumpur	The study aims to determine the attitude toward kidney donation among the public (parents of school-going students) in Kuala Lumpur.	Cross-sectional	Five schools in Kuala Lumpur	Only 22% of parents wanted to donate their kidneys. The most common reason for unwillingness to donate a kidney was ‘dislike of body parts missing after death’ (51%) followed by ‘organ donation was against their religion’ (26%) which also was the commonest reason given by Muslim participants. On the other hand, 32% of those who were willing to donate their kidneys were happy their kidneys still be useful to someone and 18% were motivated to help others. It can also be noted that respondents with higher income, higher education, and previous blood donors were more willing to donate their kidneys.
Wong; Knowledge, attitudes, practices, and behaviours regarding deceased organ donation and transplantation in Malaysia multi-ethnic society: a baseline study; (2011) (2)	Selangor	To understand participants’ knowledge, attitudes, practices, and behaviours on deceased organ donation and transplantation.	Cross-sectional	Community	There were significant differences in mean total knowledge and attitude scores among various socio-demographics. Participants who reported that they have registered to be organ donors have significantly higher mean total knowledge and attitude scores. It is shown that participants with better knowledge have a higher propensity toward organ donation. A total of 283 out of 390 respondents that have not registered as organ donors but expressed willingness to donate their organs after death, claimed that they were unsure of the ways to register as organ donors.
Loch et al.; Differences in attitude towards cadaveric organ donation: observations in a multiracial Malaysian society; (2010) (8)	Kuala Lumpur	This study aims to assess the overall knowledge and attitude about cadaveric organ transplantation among the relatives of patients awaiting treatment in the emergency department. The study also sought to identify the factors associated with unwillingness towards cadaveric organ donation.	Cross-sectional	University of Malaya Medical Center, Kuala Lumpur	Overall, 88.3% of the participants were aware of organ transplantation in general and 62.3% were aware they could donate their organs after death. Only 10% could correctly identify the definition of brain death however, 28% identified that brain death is an irreversible condition, 43.6% said they would donate their organs, 10.6% would not donate their organs and 45.8% were unsure if they want to donate their organs. The most common reason for not being willing to donate was ‘organs can be used for medical research’ (18.8%), followed by ‘would like to be buried as a whole’ (18%). In addition, 2.1% were not willing to donate their organs as ‘they did not know how to go about doing so’. Lower willingness was associated with lower educational level, Malay ethnicity, Muslim religion and older age (> 50 years old). However, there was no significant relation between knowledge of organ donation as well as brain death and willingness.
Bhandary et al.; Eye donation-awareness and willingness among attendants of patients at various clinics in Melaka, Malaysia; (2011) (27)	Melaka	The aim of the study was to assess the awareness about eye donation and willingness to donate eyes among attendants of patients at various clinics in Melaka, Malaysia.	Cross-sectional	Outpatient departments of the General Hospital and two peripheral clinics in Melaka	The majority (88%) of the participants were aware that eyes could be donated only after death and that their most common source of information was through media and newspapers. However, only one-third (34.42%) of them were willing to donate their eyes. The most common misconceptions among the participants about eye donation were that eyes could not be retrieved at the house of the deceased (90.3%) and 43.3% felt that the whole eyeball was transplanted to the patient.
Ismail et al.; Knowledge, attitude and factors influencing public willingness towards organ donation among hospital patients and relatives in Negeri Sembilan, Malaysia; (2020) (1)	Negeri Sembilan	This paper aims to assess the attitude, beliefs and knowledge of patients and relatives at three different hospitals in Negeri Sembilan towards organ donation.	Cross-sectional	Hospital Tuanku Ja’afar, Hospital Port Dickson and Hospital Tuanku Ampuan Najihah in Negeri Sembilan, Malaysia	It was found among the participants only 35% were willing to donate their organs out of which 6% had already been registered as organ donors. The most common reason for non-willingness towards organ donation was ‘family disapproval’ and ‘fear of mutilating the corpse’. Source of knowledge had a highly significant overall effect on willingness to be organ donors followed by occupation, race, religion, education and marital status. The source of information about knowledge of organ donation played a significant role as those who obtained knowledge from campaigns were 2.9 times more likely to be willing to donate compared to those who read newspapers. Also, those who were unmarried, employed and non-Muslims seem to have greater willingness towards organ donation than their counterparts.
Venorich et al.; Awareness on organ donation among urban and rural communities in Bintulu; (2020) (28)	Bintulu, Sarawak	To investigate the awareness, knowledge, and practices of organ donation among the public in Sarawak.	Cross-sectional	Community	Respondents from the urban area showed higher awareness of organ donation compared to rural areas (86.1% versus 73.9%) and 70.5% of urban respondents agreed organ donation is needed in our country. In general, urban respondents showed better knowledge of organ donation which included the definition, treatment and side effects. Urban respondents showed better practice compared to rural respondents.
Tumin et al.; Low organ donation rate in Malaysia: a survey; (2014) (10)	Klang Valley	To evaluate the willingness of Malaysians towards organ donation and identify the various reasons stated by non-willing Malaysians.	Cross-sectional	Community	Precisely 44.3% of participants were willing to donate their organs after death but only 4.3% of those willing to donate are registered organ donors. Reasons for refusal to be a donor include religious reasons, wanting their bodies to be intact after death, lack of information and lack of confidence that their organs would be used beneficially.
Tumin et al.; Muslims’ views on the permissibility of organ donation: the case of Malaysia; (2016) (17)	Kuala Lumpur	To identify Malaysian Muslims’ views on the permissibility of organ donation.	Cross-sectional	Muslim community	Precisely 55.1% of respondents do not believe that organ donation is prohibited in Islam and 58% agree that organ donation is a communal responsibility and is following Islamic principles. About half of the respondents agree that a donor and his or her family are entitled to receive a reward for donating organs or for giving consent to donate their dead relative’s organs.
Nur Idayu and Zukarnain; Knowledge, religious beliefs and perception towards organ donation from death row prisoners from the perspective of patients and non-patients in Malaysia: a preliminary study; (2012) (29)	Kuala Lumpur	To measure Malaysians’ basic knowledge of organ donation and their religious perspectives regarding organ donation.	Cross-sectional	Several hospitals in Kuala Lumpur and Klang Valley	Precisely 41.3% of non-patients and 13.6% of patients acknowledged that Malaysia is currently facing a shortage of organ donors while approximately 16% of non-patients and 9% of patients were unaware. Precisely 31.9% of non-patients and only 6.1% of patients knew the correct definition of organ donation. Most of the participants believe that their religion allows for organ donation. 32.4% of non-patients and 8.5% of patients will accept the organs donated by prisoners while 20.7% of both non-patients and patients would not. The rest, 38.5% are not sure whether to accept or not organ donation from death row prisoners and out of this 5.2% are registered patients for organ transplants. When it comes to accepting organs from death row prisoners for their close family members, we found that 48.8% of the respondents are willing to accept organs donated by death row prisoners. 6.1% of patients and 33.3% of non-patients do not mind accepting organs donated by any type of death row prisoner. 26.8% of participants would not accept organs from any type of death row prisoner, but if they were to receive organs from the prisoners, they would like to know what type of prisoners donated the organ and what kind of crime the prisoners committed before. In the case of Muslim respondents, 43.7% of both patients and non-patients are willing to donate their organs. The majority of Christians (73.3%), Buddhists (47.8%) and respondents in the ‘Other’ religion (87.5%) would also do the same.
Nur Zainie et al.; Barriers to organ donation: a study in Alor Setar, Malaysia; (2015); (30)	Kedah	To assess the influence of factors influencing organ donation decision with the willingness to donate an organ.	Cross-sectional	Community	In this study, 9.48% of participants will never consider donating an organ, 60.3% will think about donating an organ, 17.2% will only like to donate under special circumstances and 11.2% will want to donate. Some respondents believe that organ donation will harm their current health status. The perceived risks may include infections (30%), pain (20%), bleeding (19%), body weaknesses (16%) and anxiety/depression (14%). 5% of the respondents believed their organs will be misused after being taken out by the providers. Most respondents also believe that the relationship between the donor and recipient plays the most important role in organ donation.
Riyanti et al.; Organ donation by sociodemographic characteristics in Malaysia; (2014) (31)	Kuala Lumpur	To determine the proportion of those who had received information about organ donations, the proportion of those who were willing to donate after death, the factors associated with not wanting to donate, and the main reasons for refusal.	Cross-sectional	Community	In this study, the majority of the respondents (98.5%) had no intention of donating their organs after death. 69.6% of the respondents received information regarding organ donation. Almost half of the respondents (52.2%) disagree that the currently available information influenced them to pledge to donate their organs after death. Respondents with tertiary education had a significantly higher prevalence of having received information regarding organ donation and a higher prevalence of agreeing with organ donation, followed by those with secondary education. Fear was the main reason given for not pledging as organ donors followed by uncertainty because of religion and against religious practice.
Tumin et al.; Determinants of willingness to become organ donors among dialysis patients’ family members; (2015) (32)	Kuala Lumpur	To explore the factors affecting the willingness of dialysis patients’ family members to become involved in living and deceased organ donation.	Cross-sectional	Community	The findings showed that if the respondent is a child in the family, she/he is more likely to be an organ donor compared with being a spouse or a parent. Those with lower education are less likely to be organ donors compared with respondents with higher education. In the decision to become a deceased donor, we found that knowledge plays a role. As expected, the higher the knowledge, the more likely the individual is to be an organ donor. The reluctant respondents in this study (39.4%) indicated that they are ‘medically unfit’ to be organ donors. Other reasons behind their unwillingness to be living donors included being ‘scared of surgery’ (27.1%) and ‘objection by family’ (24.9%). As for the deceased donation, the most cited reason among respondents was ‘inadequate information’ (42.0%), followed by ‘objection by family’ (33.4%).

**Table 2:** Summary of included studies for Knowledge, attitude and intention to donate among healthcare personnel

Author(s); Article title; Year	State	Study aim	Study design	Study setting	Results
Abidin et al.; Are health professionals responsible for the shortage of organs from deceased donors in Malaysia?; (2012) (19)	Kuala Lumpur	1. To assess the attitudes, knowledge, and understanding of health professionals in Malaysia in organ transplantation, identifying suitable donors, and activating the organ donation process. 2. To examine the frequency of requesting organ donation to the families of potential donors.	Cross-sectional	University of Malaya Medical Centre and Hospital Kuala Lumpur	The findings demonstrate the role healthcare professionals play in the shortage of deceased organ transplantation may be affected by misconceptual recognition of brainstem death and lack of knowledge on contacting the Organ Transplant Coordinator. It was shown that healthcare professionals remain passive in approaching families of potential donors and informing the transplant teams. Of 462 respondents, 221 (47.8%) have the intention to donate their organs, with varied ethnic and religious associations.
Foong et al.; Demographics of healthcare professionals’ knowledge and attitude toward deceased organ donation: survey of critical care areas in a tertiary hospital; (2018) (21)	Kuala Lumpur	The study aims to identify the demographics of healthcare professionals working in the critical care areas and their knowledge and attitudes toward the deceased organ donation process.	Cross-sectional	Four critical care areas of Hospital Kuala Lumpur: General Medical intensive care unit (ICU), Neurosurgery ICU, Neurology department and Emergency and Trauma Unit	Most of the respondents (60.4%) were nurses. The majority of respondents were females (77.2%). Doctors had overall better knowledge about brain death, organ donation, and transplantation. Neurosurgical ICU HCP had better knowledge about brain death compared to emergency department HCPs. Regarding attitude towards brain death, doctors seem to be more convinced of brain death easily than nurses. Also, doctors seem to have a 2.71 times greater chance than nurses to accept organ transplantation for themselves.
Tumin et al.; Factors associated with Health care professionals’ attitude toward the presumed consent system; (2019) (23)	Kuala Lumpur	To explore the attitude of healthcare professionals toward the presumed consent system.	Cross-sectional	University of Malaya Medical Centre	If the presumed consent system were to be implemented, 175 (45.8%) out of 382 respondents would object to donating their organs. Findings revealed that the Malay ethnic group was more likely to object compared to other ethnicities. Healthcare professionals with an income of RM3,000 or below were more likely to object compared to that earning above RM3,000. Those who were initially unwilling to donate organs, they were more likely to object to the presumed consent system compared to those who were initially willing to donate.
Shukeri et al.; The impact of educational intervention on attitude toward organ donation among health care workers in Malaysia; (2022) (22)	Kuala Lumpur, Selangor, Pahang, Kelantan	To assess the effects of an educational intervention.	Interventional study	Universiti Kebangsaan Malaysia Medical Centre, Universiti Teknologi MARA Medical Centre, International Islamic University Malaysia Medical Centre, Universiti Sains Malysia Hospital and Tengku Ampuan Afzan Hospital	Out of 458 healthcare professionals, only 345 (75.3%) completed the tests. There was an increase in willingness for organ donation post-intervention. Healthcare professionals were more likely to support organ donation in the case that they or their relatives were diagnosed with end-stage organ failure. Overall positive attitude scores were higher in adopting organ donation as part of end-of-life care, more comfortable talking about organ donation with relatives, and so on after intervention.
Oo et al.; Knowledge and attitudes of healthcare professionals and the impact on willingness to donate organs: a tertiary hospital survey (2020); (20)	Kuala Lumpur	To measure the association between healthcare practitioners’ knowledge of brain death, organ donation, and organ transplantation and their willingness to donate.	Cross-sectional	Hospital Kuala Lumpur	According to this study, doctors were more willing to donate than nurses. Willingness to donate was significantly associated with overall knowledge score. Precisely 68.0% of HCPs expressed their willingness to donate organs. Amongst those willing to donate, only 37.3% were carrying a donor card at the time of this study. HCPs who believed that their religion did not object to deceased OD were more willing to donate. Precisely 78.9% of HCPs believe that organ transplantation, when indicated, is a good form of treatment for patients and 70.6% were willing to accept a deceased donor organ if required. 49.3% of HCPs believe that families can consent to have their relative’s organs donated after brain death has been confirmed. The majority of the respondents (85.7%) believe OD rates are low in this country because of a lack of counselling for families of patients who are certified brain dead and more than half (69.4%) of them are not sure or disagree that if a patient has pledged to donate their organs without their family’s consent, families do not have the right to refuse donation after the patient’s death.
Rozaidi et al.; The health care professional’s attitudes towards brain death and cadaveric organ transplantation; (2000) (24)	Kuala Lumpur	To study the concept of brain death, withdrawal, and discontinuation of life support in brain-dead patients, the acceptance of cadaveric organ donation and transplantation amongst Malaysian HCPs	Cross-sectional	Two tertiary hospitals in Kuala Lumpur	Precisely 49.5% of the population are willing to become cadaveric organ donors and 86% of them supported cadaveric organ/tissue donation and transplantation programmes. Religious beliefs are the major reason why most participants reject the programme while others are rejected because of their own beliefs.

**Table 3:** Summary of included studies for knowledge, attitude, and intention to donate among health science students

Author(s); Article title; Year	State	Study aim	Study design	Study setting	Results
Tumin et al.; Factors associated with medical and nursing students’ willingness to donate organs; (2016) (15)	Kuala Lumpur	To assess factors associated with medical and nursing students’ willingness to donate organs upon death.	Cross-sectional	Undergraduate medical and nursing students from the University of Malaya	Among the total of 500 medical and nursing students, 278 (55.6%) were willing to donate their organs upon death. Out of the 278 respondents, 44 held a donor cards. Non-Malay ethnicities were 1.98 times more willing to donate their organs compared to the Malay ethnicity. Medical students were 2.53 times more willing to donate compared to nursing students. Respondents who have a family member with a donor card and confirmed their religion permits organ donation, were more willing to donate their organs.
Al-Naggar and Al-Jashamy; Practice and barriers towards organ donation among young Malaysians; (2013) (33)	Selangor	The study aims to determine Management and Science University students’ practice and barriers to organ donation.	Cross-sectional	Management and Science University, Shah Alam	The main barrier to organ donation among the students was found to be the lack of knowledge. Regarding willingness, only 6.6% of them were registered as organ donors however, more than half (50.3%) of study participants expressed their desire to be organ donors in the future.
Keya et al.; The gift of life: knowledge and attitude toward organ donation among medical students in Malaysia; (2021) (18)	Kedah	To determine the knowledge and attitude toward organ donation among medical students in a private medical institution in Malaysia.	Cross-sectional	Year 4 MBBS students of AIMST University	The study shows that 54 (44.3%) out of 122 respondents had adequate knowledge about organ donation. All of them understood the meaning of brain death and knew the place of registration for organ donation. 59 (48.4%) out of 122 respondents were willing to donate their organs in the future. It was noted that younger respondents were more likely to have better knowledge about organ donation. This study showed no statistically significant relationship between knowledge of organ donation and attitude.
Bharti et al.; Awareness and knowledge on eye donation among university students; (2008) (14)	Kuala Lumpur	This study aimed to assess the awareness and knowledge of eye donation among the students studying first-year degree courses (Medicine, Dentistry, Laboratory Technology, Pharmacy, Biomedicine and Bioengineering) in the University of Malaya.	Cross-sectional	University of Malaya	Most of the participants (86%) were aware about the existence and practice of eye donation and the knowledge was acquired through mass media, TV, radio or movies. However, only 27% of students were keen to pledge their eyes for donation, while 33% of them were willing to donate their close relative’s eyes. Overall the knowledge and awareness about eye donation were poor among the students and the governmental and non-governmental organizations must spread awareness to improve the knowledge among the public so that the eye organ transplantation rates would improve in Malaysia.
Haque et al.; Organ donation and transplantation: awareness, attitude, and aptitude of the UniKL-RCMP students, Malaysia; (2015) (13)	Perak	The objective of the study was to identify the level of knowledge, attitude, and commitment among the students of Universiti Kuala Lumpur - Royal College of Medicine Perak towards organ donation.	Cross-sectional	Universiti Kuala Lumpur -Royal College of Medicine Perak	The majority of the participants (73%) had good knowledge and 27% had average knowledge. Regarding attitude, more than half (58%) of the participants had a good attitude, 41% had an average attitude and 0.6% had a poor attitude. It was found that the MBBS students had better knowledge and attitude towards organ donation compared to students of diploma of radiology and diploma of pharmacy. However, the students of diploma of pharmacy had better commitment among the 3 groups of students. Overall, 55% of them had an average commitment, 41% and 5% had poor and good commitment regarding organ donation, respectively.
Huern et al.; Knowledge, awareness and attitudes on organ donations among undergraduate medical students in Malaysia: an analytical cross-sectional study; (2016) (34)	Melaka	i) To assess the knowledge, attitudes and perception about organ donation. As well as to determine the prevalence of organ donors among private undergraduate medical students in Malaysia. ii) To establish a relationship between various socio-demographic data on knowledge, awareness and perception of organ donation among the students.	Cross-sectional	Melaka Manipal Medical College (MMMC), Melaka, Malaysia	Only about 14.5 % of the students were registered organ donors however 71.2 % of students were willing to register as organ donors in the future and the majority of these were Indian and Chinese students. Good knowledge corresponded to increased willingness. Older age, males, Indians and students from other religions (apart from Hinduism, Buddhism, Muslim and Christians) had better knowledge compared to others. Also, regarding awareness, 77.7% of all the students were aware of the organ donation registry. Students from other religions and those whose parents had higher educational qualifications seem to have a better awareness.
Jun; Allied Health Sciences College students’ perception towards organ donation and determinant towards the intention to pledge: evidence from Sarawak; (2019) (16)	Sarawak	This study assesses the perception according to diverse ethnic communities, towards kidney donation and determine the factors contributing to the intention to pledge kidney among the Semesters 5 and 6, Allied Health Sciences Diploma students (Nursing, Medical Assistant and Medical Laboratory Technology) in ILKKM Kuching, Sarawak.	Cross-sectional	Institut Latihan Kementerian Kesihatan Malaysia (ILKKM) Kuching, Sarawak	The knowledge of the majority of the participants was of medium level. The participants also showed a positive attitude and 74.6% were aware of the existence of an organ donation registry. However, only 50.7 % of the participants were willing to donate their organs and 49.3% of the were not willing to do so. The most common reason given by the participants was lack of information and not having decided of becoming a pledger. Having a father with higher educational qualifications added to a better attitude and willingness toward organ donation among the participants.
